# Supplementary material for: Time‑resolved multi-omic analysis of paclitaxel exposure in human iPSC‑derived sensory neurons unveils mechanisms of chemotherapy‑induced peripheral neuropathy
Source: Cell Death Dis. 2026 Feb 10;17(1):211. doi: 10.1038/s41419-026-08445-2 (PMC12921266; doi:10.1038/s41419-026-08445-2)
Supplement: Supplementary file 15 — Dataset 1. [file 41419_2026_8445_MOESM15_ESM.zip › CDD_Open_Data/04_RNA_Seq/Timeline_panel_plots_etc_2023-01-23_1609.zip!/Panel_Plots_etc/Evidence_Plots/72h/evidence_plot_standard contrasts_msigdb_hallmark_72h_PTX_vs_DMSO_ID0_M5892_HALLMARK_CHOLESTEROL_HOMEOSTASIS.pdf]

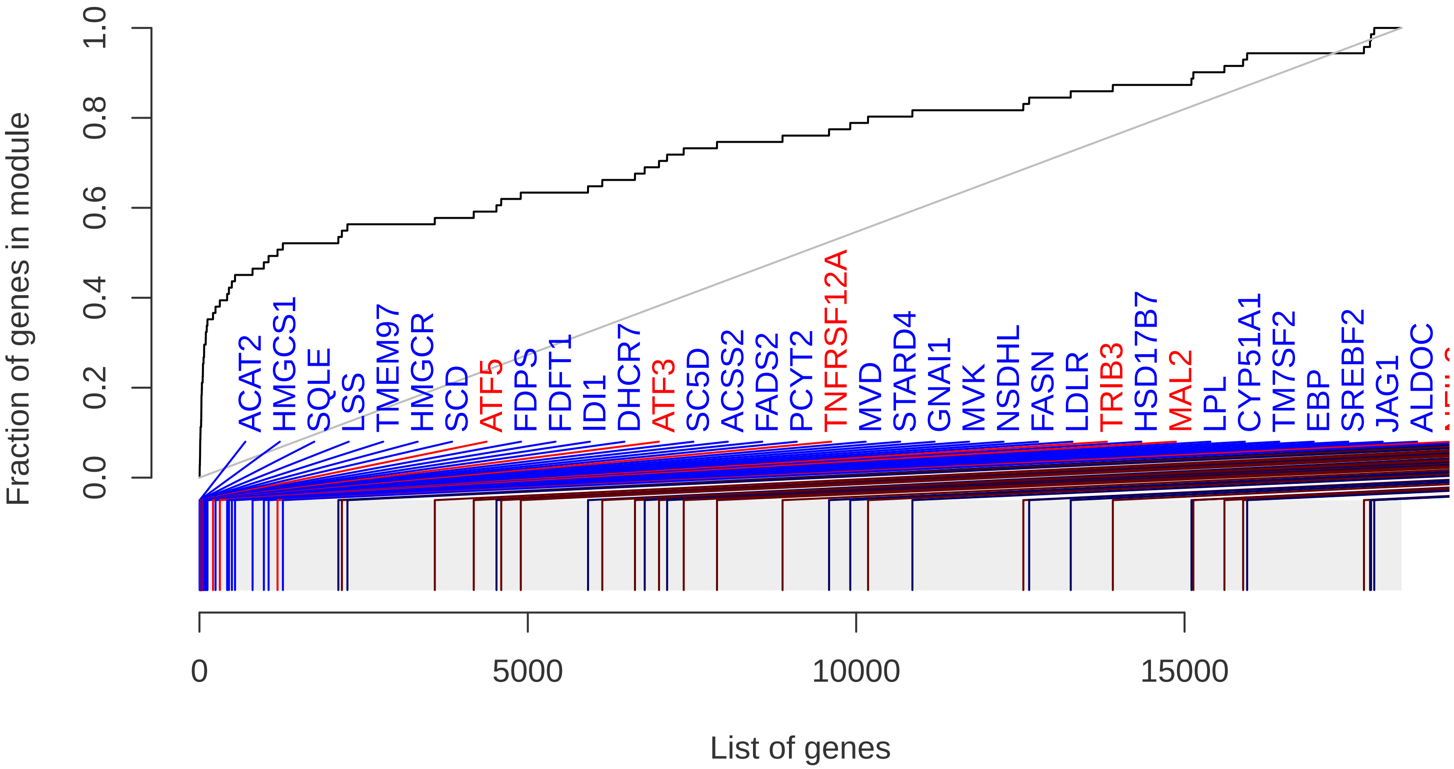

Fraction of genes in module
0.0
0.2
0.4
0.6
0.8
1.0
ACAT2
HMGCS1
SQLE
LSS
TMEM97
HMGCR
SCD
ATF5
FDPS
FDFT1
IDI1
DHCR7
ATF3
SC5D
ACSS2
FADS2
PCYT2
TNFRSF12A
MVD
STARD4
GNAI1
MVK
NSDHL
FASN
LDLR
TRIB3
HSD17B7
MAL2
LPL
CYP51A1
TM7SF2
EBP
SREBF2
JAG1
ALDOC
0
5000
10000
15000
List of genes
